# Reliability and Validity of the Self-Report Chinese Version of the Yale-Brown Obsessive–Compulsive Scale Modified for Body Dysmorphic Disorder (BDD-YBOCS) in Patients Undergoing Plastic Surgery

**DOI:** 10.1007/s00266-022-02784-z

**Published:** 2022-02-24

**Authors:** Xiaodong Chen, Gang Chen, Jinming Wang, Jinlong Huang

**Affiliations:** Department of Plastic and Reconstructive Surgery, Jiangsu Province Hospital of Chinese Medicine, Affiliated Hospital of Nanjing University of Chinese Medicine, 155 Hanzhong Road, Nanjing, 210029 China

**Keywords:** BDD-YBOCS, Reliability, Validity, Plastic surgery, Self-report scale

## Abstract

**Background:**

To develop a self-report Chinese version of the Yale-Brown Obsessive–Compulsive Scale Modified for Body Dysmorphic Disorder (BDD-YBOCS) and determine its validity and reliability in patients seeking a consultation with a plastic surgeon or undergoing plastic surgery in China.

**Methods:**

Forward and backward translation and cultural adaptation of the BDD-YBOCS were performed according to recommended guidelines. The self-report Chinese version of the BDD-YBOCS was psychometrically tested using data collected from a cross-sectional validation study, which included 240 patients seeking a consultation with a plastic surgeon or undergoing plastic surgery at the Department of Plastic Surgery, Jiangsu Province Hospital of Chinese Medicine, Nanjing, China, between May and September 2020. Item analysis used the independent sample *t* test and bivariate Pearson test. Content validity was established through expert interviews. Construct validity was measured with exploratory factor analysis (EFA) and confirmatory factor analysis (CFA). Convergent validity and discriminant validity were analyzed using Pearson’s correlation to evaluate the association between the self-report Chinese version of the BDD-YBOCS and the Body Dysmorphic Disorder Questionnaire (BDDQ). Internal reliability was assessed using Cronbach’s α.

**Results:**

No items were removed from the original English version of the BDD-YBOCS based on expert interviews and factor analysis. A total of 220 patients completed the study survey (91.7%). EFA extracted 3 factors, which accounted for 64.50% of the variance. CFA supported a 3-factor structure (*χ*^2^/*df* = 1.322, RMSEA = 0.054, GFI = 0.904, NFI = 0.902, CFI = 0.974 and TLI = 0.966). The scale had good convergent and discriminant validity. Cronbach’s α for the scale was 0.871 (*P* < 0.001) and ranged from 0.852 to 0.873 when individual items were removed.

**Conclusion:**

The self-report Chinese version of the BDD-YBOCS shows good validity and reliability for use in patients seeking a consultation with a plastic surgeon or undergoing plastic surgery in China.

**Level of Evidence IV:**

This journal requires that authors assign a level of evidence to each article. For a full description of these Evidence-Based Medicine ratings, please refer to the Table of Contents or the online Instructions to Authors www.springer.com/00266.

## Introduction

According to the Diagnostic and Statistical Manual of Mental Disorders V (DSM-V) [1], body dysmorphic disorder (BDD) is an obsessive–compulsive spectrum disorder. DSM-V defines BDD as a mental health condition characterized by a distressing or impairing preoccupation with an imagined or a slight defect in one’s appearance. BDD represents an intersection between surgery and psychiatry, as individuals with BDD may seek cosmetic or plastic surgery due to an exaggerated dissatisfaction with the appearance of their body, when in actuality, a psychiatrist should treat them [2]. Due to limited research on BDD [3], many cosmetic and plastic surgeons cannot recognize and accurately diagnose patients with BDD. A cosmetic or plastic surgeon may provide treatment without realizing a patient is unsuitable for the procedure. Patient dissatisfaction with the outcome may result in legal action or physical violence against the surgeon [4–6].

The prevalence of BDD in the general population is estimated at 0.7–13% [7–10]. Specifically, BDD epidemiological surveys conducted in 2004indicated that the prevalence of DSM-IV BDD among respondents in Germany was 1.7% (*n* = 42/2552) (95% CI 1.2–2.1%), and higher in women (1.9%) than men (1.4%) [9], and 2.4% (49/2048) in the USA (2.5% for women, 2.2% for men) [10]. Previous studies have shown that BDD is more common in patients seeking plastic surgery and those attending dermatology clinics than in the asymptomatic general population [11, 12]. A meta-analysis revealed that 15.04% (range 2.21–56.67%) of patients undergoing plastic surgery had BDD; patient mean age was 34.54 ± 12.41 years, and the majority of patients were women (74.38%). Among dermatology patients, 12.65% (range 4.52–35.16%) had BDD; patient mean age was 27.79 ± 9.03 years, and 76.09% were women [11]. It is essential to raise awareness about BDD among cosmetic and plastic surgeons, as they are likely to encounter patients with BDD in their practice. Cosmetic and plastic surgeons must be familiar with BDD and able to refer patients to a psychiatrist for diagnosis and appropriate management.

The Yale-Brown Obsessive–Compulsive Scale Modified for Body Dysmorphic Disorder (BDD-YBOCS) is a 12-item, semi-structured, rater-administered measure that assesses BDD severity during the past week [13]. The scale is widely used, has good validity and reliability, and is sensitive to change in individuals seeking a clinical evaluation or treatment for BDD. The BDD-YBOCS is available as an English, Thai [14], or Brazilian [15] translation.

The validity and reliability of the BDD-YBOCS in patients undergoing plastic surgery in China have not been assessed, and currently, there is no screening tool for BDD in China. The objective of this study was to translate and culturally adapt the BDD-YBOCS to a self-report Chinese version and determine its validity and reliability in patients seeking a consultation with a plastic surgeon or undergoing plastic surgery in China.

## Subjects and Methods

### Participants

Patients seeking a consultation with a plastic surgeon or undergoing plastic surgery at the Department of Plastic Surgery, Jiangsu Province Hospital of Chinese Medicine, Nanjing, China, between May and September 2020, were eligible for this study. Inclusion criteria were: (1) age > 18 years; (2) attending our hospital seeking a consultation with a plastic surgeon or undergoing plastic surgery; (3) voluntarily willing to participate in the study; and (4) able to complete the study survey. Exclusion criteria were (1) inability to understand the study survey; (2) severe physical deformities resulting from tumors or other conditions; or (3) mental disorders such as schizophrenia, depression, or phobia.

The study was conducted according to the ethical standards of the institutional and national research committees and the 1964 Declaration of Helsinki and its later amendments or comparable ethical standards. All patients provided written informed consent to participate in the study. Researchers were trained on how to administer the questionnaires and collect data.

### Evaluations

#### BDD-YBOCS

The BDD-YBOCS is a 12-item semi-structured clinician-rated scale designed to measure BDD symptom severity. The first five items assess excessive obsessional preoccupations with perceived appearance defects, including time preoccupied, interference and distress due to the preoccupations, resistance against the preoccupations, and control over the preoccupations. Items 6 through 10 assess compulsive behaviors, such as excessive grooming and mirror checking. Items 11 and 12 assess insight into appearance beliefs and avoidance due to BDD symptoms, respectively. Each item is scored on a 5-point scale, ranging from 0 (not affected) to 4 (extremely affected). A total score is calculated as the sum of the scores for each individual item, for a maximum of 48. The BDD-YBOCS has demonstrated good validity and reliability among patients undergoing cosmetic or plastic surgery and is the most widely used measure in studies evaluating the efficacy of treatments for BDD [13].

#### Body Dysmorphic Disorder Questionnaire (BDDQ)

The BDDQ is a brief 5-item self-administered questionnaire based on the DSM-IV diagnostic criteria for BDD [16]. The first question establishes whether preoccupations are present. If the answer is yes, respondents continue to Questions 2 and 3, which assess distress and impairment caused by the preoccupations (distress, social life, work/school, or avoidance). Question 4 assesses time spent on thinking about the perceived defects, with respondents likely to have BDD if they spend > 1 hour a day thinking about how they look. Question 5 could indicate the presence of either BDD or an eating disorder, requiring evaluation by a clinician to ensure an accurate diagnosis. The Chinese translation of the BDDQ has been validated in a sample of patients undergoing plastic surgery (*n* = 222). It exhibited a sensitivity of 100% and a specificity of 93%, and the correlation coefficient of each item was between 0.808 and 1.000 (*P* < 0.001). This suggests the Chinese translation of the BDDQ has high validity and reliability [17].

### Translation and Cross-cultural Adaptation of the BDD-YBOCS

Forward and backward translation and cultural adaptation of the BDD-YBOCS were performed according to recommended guidelines to develop a self-report Chinese version of the BDD-YBOCS [18].

Two independent bilingual translators with Master’s degrees and experience in psychology or plastic surgery created Chinese translations of the BDD-YBOCS and consolidated them into a single version after discussion. Two independent bilingual native Chinese translators, one with a doctorate in statistics from the USA and the other a professional English translator, back-translated the consolidated version from Chinese to English. The translated and original versions were reviewed to ensure translated items retained the meaning of the original items, without confusion.

The Chinese translation of the BDD-YBOCS was revised into a self-report scale by two professors from the Psychology Department, who modified wording and provided notes. The revised scale retained the 12 items included in the original scale, but items were reordered. General information on the respondents was collected, including gender, age, marital status, highest education, occupation, and history of plastic surgery. An expert committee comprised of 10 native Chinese-speaking experts in the fields of plastic surgery and psychology evaluated the cultural aspects and language of the self-report Chinese version of the BDD-YBOCS and proposed modifications. Items marked as “unclear” by over 20% of the committee participants were revised. The clarity of the items in a pre-final version was pilot tested in 20 patients. Of these, 8 patients misunderstood item 11 and its anchors (*“Is it possible that your defect might be less noticeable or less unattractive than you think it is?” scored as 0=excellent insight, 1=good insight, 2=fair insight, 3=poor insight, and 4=absent insight*); therefore, the anchors were replaced with: *0=yes, very much possible, 1=possibly yes, 2=not sure, 3=possibly not, 4=no, definitely not possible*.

### Statistical Analyses

Statistical analyses were performed with SPSS v23.0 and Amos v23.0. *P* < 0.05 was considered statistically significant. Descriptive statistics, means, and standard deviations were used to summarize patients’ demographic data. Psychometric evaluation of the self-report Chinese version of the BDD-YBOCS was performed through item analysis, and measurement of content validity, construct validity, convergent validity, discriminant validity, and internal reliability.

Item analysis used the independent sample *t* test to compare patients with high scores and low scores on the self-report Chinese version of the BDD-YBOCS and the bivariate Pearson test to calculate the item-total correlation. Content validity was established through expert interviews. Construct validity was measured with exploratory factor analysis (EFA) and confirmatory factor analysis (CFA). Convergent validity and discriminant validity were analyzed using Pearson’s correlation to evaluate the association between the total score on the self-report Chinese version of the BDD-YBOCS and the BDDQ. Internal reliability was assessed using Cronbach’s α.

## Results

### Participants Characteristics

A total of 240 patients seeking a consultation with a plastic surgeon or undergoing plastic surgery were invited to participate in this study. Of these, 12 patients declined to join the study, and 8 patients did not complete the study survey. Finally, data from 220 (91.7%) patients were included in the analyses.

The sociodemographic and clinical characteristics of the included patients are summarized in Table 1. Mean age of the patients was 28.59 ± 9.69 years (range 18–57 years), and more than half of the patients were aged < 35 years (78.2%). Most of the patients were women (88.2%), unmarried (65.5%), or had completed higher education (78.2%). The distribution of occupation and income across the study population was relatively uniform. 19.1% of patients had undergone previous plastic surgery. Most patients (75%) were dissatisfied with the appearance of their eyes or nose. Differences in patient age, gender, marital status, occupation, and monthly income were unlikely to affect responses on the study survey, but differences in history of previous plastic surgery and self-perceived appearance may have influenced outcomes.

**Table 1:** Sociodemographic and clinical characteristics of the included patients

Characteristics		N	(%)	F	*P*
Gender	Male Female	32 194	11.8 88.2	0.001	0.097
Age	18–25 26–35 36–45 46–55 >56	92 66 32 14 2	47.2 30.0 14.5 6.4 0.9	1.049	0.386
Marital status	Unmarried Married Divorced	144 72 4	65.5 32.7 1.8	0.208	0.813
Education	Primary Secondary Higher	4 44 172	1.8 20.0 78.2	0.928	0.399
Occupation	Student Civil servant White-collar Blue-collar Unemployed	74 72 48 6 20	33.6 32.7 21.8 2.7 9.1	1.434	0.228
Monthly income	0 <3000 3000–5000 5000–8000 8000–15000 >15000	44 36 48 44 34 14	20.0 16.4 21.8 20.0 15.5 6.4	0.350	0.881
Plastic surgery history	Yes No	42 178	19.1 80.9	9.377	0.003
Appearance concerns	Eye Nose Body shape Breast Anti-aging Face shape Other	148 18 6 2 16 12 18	67.3 8.2 2.7 0.9 7.3 5.5 8.2	5.769	<0.001

### Item Analysis

Item analysis computed the critical ratio and the item-total correlation. Respondents with the highest and lowest scores on the self-report Chinese version of the BDD-YBOCS were separated into upper and lower groups comprised of the top 27% and bottom 27% of the scores. Mean total score of the upper group was 18.63 (SD = 1.67), and mean total score of the lower group was 4.1 (SD = 0.35). On the independent sample *t* test, the differences between the scores for each item in the upper and lower groups were significantly different (*P* < 0.01), indicating all items provide good discrimination. The bivariate Pearson test showed a significant positive correlation between the score for each item and the total score for the self-report Chinese version of the BDD-YBOCS (*r* = 0.598–0.765; *P* < 0.01), indicating all items are closely associated with the scale.

### Validity

#### Content Validity and Criterion Validity

Ten experts were invited to independently assess the content validity of the self-report Chinese version of the BDD-YBOCS. The item content validity index (ICVI) was 0.90 (criteria: > 0.78), indicating that the scale accurately represents all facets of the construct BDD. The total score of the self-report Chinese version of the BDD-YBOCS was significantly correlated with the total score of the BDDQ (*r* = 0.557, *P* < 0.01), indicating good criterion validity.

### Construct Validity

#### Exploratory Factor Analysis (EFA)

Before performing factor analysis, the Kaiser–Meyer–Olkin test (KMO) and Bartlett’s sphericity test were conducted to measure sampling adequacy (criteria: KMO > 0.6, Bartlett’s test *P* < 0.05). Results indicated that the sample size (*n* = 110) was adequate (KMO = 0.865, *df* = 582.58.12, *P* < 0.0001).

Principal component analysis and varimax rotation extracted 3 factors, which accounted for 64.50% of the variance (criteria: >50%). Factor 1, “obsessions,” was strongly associated with items 1, 2, 3, 4, and 5 (loadings ranged from 0.642 to 0.776) and accounted for 28.698% of the variance. Factor 2, “compulsions,” was strongly associated with items 6, 7, 8, 9, and 10 (loadings ranged from 0.551 to 0.793), and accounted for 21.463% of the variance. Factor 3, “additional,” was strongly associated with items 11 and 12 (loadings 0.809 and 0.596), and accounted for 14.335% of the variance. All factor loadings were > 0.50, suggesting that the three factors are related and are elements of the construct BDD (Table 2).

**Table 2:** Factor and item loadings and variance contribution rates of each factor

Factor	Item	Factor loading	Variance contribution rate (%)
Obsessions	Q1 Q2 Q3 Q4 Q5	0.776 0.774 0.764 0.642 0.699	28.698
Compulsions	Q6 Q7 Q8 Q9 Q10	0.600 0.551 0.619 0.793 0.783	21.463
Additional	Q11 Q12	0.809 0.596	14.335

#### Confirmatory Factor Analysis (CFA)

CFA in an independent sample (*n* = 110) supported a 3-factor structure for the self-report Chinese version of the BDD-YBOCS, with factor loadings ranging from 0.54 to 0.83. Model fit indices were *χ*^2^/df = 1.322 (criteria: < 3), CFI = 0.974, GFI = 0.904, NFI = 0.902, TLI = 0.966 (criteria for all > 0.9) and RMSEA=0.054 (criteria: < 0.08) (Table 3), indicating a good fit.

**Table 3:** The result of CFA (*N*=110)

*χ*^2^/df	RMSEA	GFI	NFI	CFI	TLI
1.322	0.054	0.904	0.902	0.974	0.966

Composite reliability (CR) considering factor loadings was used to assess internal consistency in scale items. A higher CR value represents a higher internal consistency of the factor, with 0.7 as the acceptable threshold. The CR of each factor was greater than 0.776 (Table 4).

**Table 4:** Factor loadings and convergent validity

	Estimate		
	Std	CR	AVE
Q1<---obsessions Q2<---obsessions Q3<---obsessions Q4<---obsessions Q5<---obsessions	0.794 0.799 0.832 0.660 0.654	0.865	0.565
Q6<---compulsions Q7<---compulsions Q8<---compulsions Q9<---compulsions Q10<---compulsions	0.806 0.814 0.755 0.715 0.541	0.851	0.537
Q11<---additional Q12<---additional	0.788 0.804	0.776	0.634

Convergent validity was assessed using the average variance extracted (AVE) (criterion > 0.5). AVE values for the factors demonstrated acceptable convergent validity (Table 4).

Discriminant validity was assessed by comparing the square root of AVE of each factor with correlations between pairs of factors (Fornell and Larcker 1981). The square root of AVE was larger than the correlation between any pair of factors representing satisfactory discriminant validity (Table 5).

**Table 5:** Discriminant validity

	Obsessions	Compulsions	Additional
Obsessions	0.565		
Compulsions	0.054***	0.537	
Additional	0.05***	0.045***	0.634
$\sqrt {AVE}$	0.818	0.763	0.800

### Reliability

Cronbach’s α for the self-report Chinese version of the BDD-YBOCS and the obsessions, compulsions, and additional factors were 0.871, 0.824, 0.797, and 0.637 (criteria: Cronbach’s α > 0.70), respectively, with Cronbach’s α ranging from 0.852 to 0.873 when individual items were deleted. The deletion of any item did not increase the Cronbach’s α sufficiently to imply the self-report Chinese version of the BDD-YBOCS is better without that item. Corrected item-total correlations (ITC) ranged from 0.363 to 0.707 (Table 6).

**Table 6:** Cronbach’s α and ITC

Item	α if item deleted	Corrected item-total correlation	Factor	Cronbach’s α
Q1	0.855	0.659	Obsessions	0.824
Q2	0.854	0.689		
Q3	0.852	0.698		
Q4	0.872	0.424		
Q5	0.856	0.627		
Q6	0.857	0.640	Compulsions	0.797
Q7	0.853	0.707		
Q8	0.856	0.641		
Q9	0.870	0.434		
Q10	0.865	0.513		
Q11	0.873	0.363	Additional	0.637
Q12	0.863	0.512		

## Discussion

In this study, forward and backward translation and cultural adaptation of the BDD-YBOCS were performed according to recommended guidelines to develop a self-report Chinese version of the BDD-YBOCS. The scale was psychometrically tested using data collected from a cross-sectional validation study involving 240 patients seeking a consultation with a plastic surgeon or undergoing plastic surgery in China. Findings showed the self-report Chinese version of the BDD-YBOCS has acceptable validity and reliability, representing a tool that may be used to screen for BDD and evaluate the efficacy of treatments for BDD in patients undergoing plastic surgery in China.

Item analysis of the self-report Chinese version of the BDD-YBOCS involved calculating the critical ratio (*P* < 0.01) and the item-total correlation (*r* = 0.598–0.765, *P* < 0.01). Results confirmed that no items are redundant and should be removed from the tool. In pilot testing, most patients understood the items and answered all items within 5–10 minutes. The anchors for item 11 *“Is it possible that your defect might be less noticeable or less unattractive than you think it is?”*, which assesses insight and distress, were amended to measure “self-belief” and be applicable to a self-report format.

The ICVI of the self-report Chinese version of the BDD-YBOCS was 0.90 (criteria: > 0.78), indicating that the scale has good content validity. To date, the BDDQ is the only instrument that has been translated and validated to measure dissatisfaction with physical features and facilitate screening and diagnosis of BDD in China [17]. The strong correlation (*r* = 0.557, *P* < 0.01) observed between the self-report Chinese version of the BDD-YBOCS and the BDDQ indicates that both instruments can identify the obsessive and compulsive behaviors that characterize BDD.

Factor analysis is a standard method to evaluate construct validity and test whether a scale effectively measures a construct of interest. EFA and CFA are two frequently used forms of factor analysis. Both are based on general factor models, but have differences in methodology and scale evaluation. Psychometric testing of the original version of the BDD-YBOCS included EFA, which showed three factors (DSM-IV criteria for BDD plus interference due to compulsions, insight, and avoidance; compulsions; resistance and control of thoughts) accounted for 59.6% of the variance [13]. Further examination of the psychometric properties of the original version of the BDD-YBOCS used principal components factor analysis, which identified two factors (all items except for interference due to thoughts and avoidance; avoidance and “core DSM-V” symptoms) that accounted for 66% of the variance [19]. In our study, three factors with eigenvalues > 1 and loadings > 0.5 accounted for 64.5% of the variance. The differences in the number of factors between the original and self-report Chinese version of the BDD-YBOCS may be explained by scale format and the use of disparate statistical methods. Our data indicate the self-report Chinese version of the BDD-YBOCS has good construct validity. CFA in an independent sample supported a 3-factor structure, with factor loadings > 0.50. CR provided evidence of good internal consistency. AVE values for the factors demonstrated acceptable convergent validity, and the square root of the AVE values was larger than the correlation between any pair of factors representing satisfactory discriminant validity.

Internal consistency reliability verified that the self-report Chinese version of the BDD-YBOCS addresses the construct of BDD (Cronbach’s *α* = 0.871), and the factors obsessions, compulsions, and additional yielded similar scores (Cronbach’s α 0.824, 0.797, and 0.637, respectively).

### Study Limitations

The present study had some limitations. First, psychometric testing of the self-report Chinese version of the BDD-YBOCS was performed using data collected from patients seeking a consultation with a plastic surgeon or undergoing plastic surgery at one institution, leading to the potential for selection bias. Future studies require larger sample sizes across multiple institutions to ensure data are generalizable to the population in China. Second, we developed a self-report version of the BDD-YBOCS. Self-report measures are limited by response biases and constraints on self-knowledge. We recommend that our version of the BDD-YBOCS is used for preliminary screening of possible cases of BDD. Third, our evaluation of the criterion validity of the self-report Chinese version of the BDD-YBOCS was restricted to a comparison with one tool, the BDDQ. Fourth, plastic surgeons used subjective judgment to determine whether patients met the exclusion criterion for a pre-existing mental disorder. Patients that were considered “not quite right” were referred to the psychiatric department for further evaluation. This method of identifying pre-existing mental disorders may have caused some potentially eligible patients to be excluded from the study. Last, we did not determine the sensitivity and specificity of the self-report Chinese version of the BDD-YBOCS or identify cutoff points for symptom severity.

## Conclusion

The current study suggests that the self-report Chinese version of the BDD-YBOCS is a valid and reliable instrument to assess BDD in patients seeking a consultation with a plastic surgeon or undergoing plastic surgery in China. It may help researchers screen and evaluate interventions aimed at reducing the symptoms of BDD. Future research is needed to further evaluate the instrument and support its application in China.

## Appendix

### Self-report Chinese version of the BDD-YBOCS

**Table:**

**中文版躯体变形障碍严重程度等级量表**
1. 您每天花在考虑外貌缺陷或不足的时间是?
2. 这些想法多大程度上影响了您的社交和工作? (有因此而不想做或不能做的事情吗?)
3. 这些想法引起您多大程度的痛苦? (例如让您感到不安或焦虑)
4. 您在努力抗拒这些想法吗? (即当有这些想法时, 您努力尝试不去想它们或转移注意力, 不管是否能成功。)
5. 您是否能成功控制这些想法或成功转移注意力?
关于以上外貌缺陷或不足的想法, 您有哪些与之相关的行为,请勾选:
6. 以上行为每天占用您多少时间?
7. 这些行为在多大程度上影响了您的社交或工作? (是否因此而不能完成一些事情?)
8. 如果这些行为被阻止, 您感到焦虑或不安的程度?
9. 您在努力抗拒这些行为吗? (即您试图减少这些行为的次数)
10. 执行这些行为的驱动力 (愿望) 有多强?您能否成功控制这些行为?
11. 关于您的外貌缺陷或不足的想法, 是否有可能并没你认为的那么严重? (即您认为很重, 而别人认为很轻微或正常)
12. 您是否会因为以上想法或行为而回避做某些事情、去某些地方、或与某些人在一起?

## Appendix

### Self-report Chinese version of the BDD-YBOCS

**Table:**

1. How much of your time is occupied by THOUGHTS about a defect or flaw in your appearance?
2. How much do your THOUGHTS about your body defect(s) interfere with your social or work (role) function? (Is there anything you aren’t doing or can’t do because of them?)
3. How much distress do your THOUGHTS about your body defect(s) cause you? *Rate “disturbing” feelings or anxiety that seem to be triggered by these thoughts, not general anxiety or anxiety associated with other symptoms.*
4. How much of an effort do you make to resist these THOUGHTS? How often do you try to disregard them or turn your attention away from these thoughts as they enter your mind? *Only rate effort made to resist, NOT success or failure in actually controlling the thoughts.*
5. How much control do you have over your THOUGHTS about your body defect(s)? How successful are you in stopping or diverting these thoughts?
*Read list of activities below to determine which ones the patient engages in.*
6. How much time do you spend in ACTIVITIES related to your concern over your appearance?
7. How much do these ACTIVITIES interfere with your social or work (role) functioning? (Is there anything you don’t do because of them?)
8. How would you feel if you were prevented from performing these ACTIVITIES? How anxious would you become? *Rate degree of distress/frustration patient would experience if performance of the activities was suddenly interrupted.*
9. How much of an effort do you make to resist these ACTIVITIES?
*Only rate effort made to resist, NOT success or failure in actually controlling the activities.*
10. How strong is the drive to perform these behaviors? How much control do you have over them?
11. Is it possible that your defect might be less noticeable or less unattractive than you think it is? How convinced are you that is as unattractive as you think it is? Can anyone convince you that it doesn’t look so bad?
12. Have you been avoiding doing anything, going any place, or being with anyone because of your thoughts or behaviors related to your body defects?

## Abbreviations

BDD: Body dysmorphic disorder
BDD-YBOCS: Yale-brown obsessive–compulsive scale modified for body dysmorphic disorder
DSM-V: Diagnostic and statistical manual of mental disorders V
YBOCS: Yale-brown obsessive–compulsive scale
BDDQ: Body dysmorphic disorder questionnaire
EFA: Exploratory factor analysis
CFA: Confirmatory factor analysis
ICVI: Item content validity index
KMO: Kaiser–Meyer–Olkin test
CR: Construct reliability
AVE: Average variance extracted
RMSEA: Root mean square error of approximation
GFI: Goodness-of-fit index
NFI: Normed fit index
CFI: Comparative fit index
TLI: Tucker–Lewis index
ITC: Item-total correlation
